# Changes in Vertical Jump Parameters After Training Unit in Relation to *ACE*, *ACTN3*, *PPARA*, *HIF1A*, and *AMPD1* Gene Polymorphisms in Volleyball and Basketball Players

**DOI:** 10.3390/genes16030250

**Published:** 2025-02-21

**Authors:** Miroslav Vavak, Iveta Cihova, Katarina Reichwalderova, David Vegh, Ladislava Dolezajova, Miroslava Slaninova

**Affiliations:** 1 Department of Track and Field and Sport Conditioning, Faculty of Physical Education and Sport, Comenius University Bratislava, Nábr. arm. gen. L. Svobodu 9, 814 69 Bratislava, Slovakia; 2 Department of Genetics, Faculty of Natural Sciences, Comenius University Bratislava, Mlynska dolina Ilkovicova 6, 842 15 Bratislava, Slovakia

**Keywords:** genetics, polymorphisms, vertical jump, training adaptation

## Abstract

Background/objectives: The study aims to investigate potential differences in vertical jump performance between elite basketball and volleyball players before and after a standard training session, in comparison to a control group from the general population. The analysis focuses on the influence of selected gene polymorphisms that may contribute to variations in the assessed performance parameters. Aims: The aim was to investigate the influence of *ACE* (rs4646994), *ACTN3* (rs1815739), *PPARA* rs4253778, *HIF1A* (rs11549465), and *AMPD1* (rs17602729) genes polymorphisms on the combined effects of post-activation potentiation (PAP), post-activation performance enhancement (PAPE), and general adaptation syndrome (GAS), as reflected in vertical jump performance, in elite basketball and volleyball players compared to a control group from the general population. Methods: The effects of PAP at the beginning of the training load (acute exercise), and the combined influences of PAPE and GAS following the training load were evaluated using parameters measured by the OptoJump Next^®^ system (Microgate, Bolzano, Italy). Results: A statistically significant (h, *p* < 0.05) negative effect of the CT genotype of the *AMPD1* gene on jump height was observed in the group of athletes. The CT genotype of the *AMPD1* gene negatively impacted on PAPE and GAS adaptive responses (ΔP, Δh, *p* < 0.001) also in the control group. A positive effect on the power during the active phase of the vertical jump was identified for the II genotype of the *ACE* gene and the Pro/Ser genotype of the *HIF1A* gene, both exclusively in the control group (ΔP, *p* < 0.05). Conclusion: Our findings demonstrate that different gene polymorphisms exert variable influences on the combined effects of PAPE and GAS, as reflected in vertical jump parameters, depending on the participants’ level of training adaptation.

## 1. Introduction

Genetics plays a significant role in the determination of sporting abilities [1,2]. According to De Moor et al. [3], this accounts for 66% of sports variance, where different gene polymorphisms and their combinations can largely predict and determine an athlete’s abilities. Many genes related to motor abilities have recently been described (*ACE*, *ACTN3*, *AMPD1*, *PPARA*, *PPARD*, *PPARGC1A*), but connections between genes and sports performance remain unclear [4,5,6,7,8,9,10,11,12,13,14]. High-quality sports performance is inherently multifactorial and must be executed within a precisely determined timeframe, necessitating an in-depth understanding of musculoskeletal system activation [15,16].

Elite players, including national team members, represent a specialized subset of the general population. Volleyball and basketball share similarities in playing area dimensions, match durations, and training sessions. Players in these sports often exhibit comparable somatotypes and levels of movement skill development [17,18]. The volume, intensity, and nature of the training loads are also similar, facilitating relatively easy transitions between the two sports at a young age. In both basketball and volleyball, explosive power is a critical movement ability, particularly for vertical jumping and acceleration speed. Training in these sports focuses heavily on developing these attributes.

A special component of speed-strength performance is the acute increase in strength, which is most pronounced in muscles with a high proportion of type II fibers and is detected minutes after high-intensity muscle contractions [19,20]. This phenomenon, known as Post-Activation Potentiation (PAP), enhances muscle contractile response following an intense voluntary contraction. PAP is defined as an enhanced muscle contractile response after intense voluntary contraction, measured by the maximal force of contractions elicited by supramaximal electrical stimulation [21].

Performance in the vertical jump with counter movement (CMJ) can be influenced additionally by high-intensity activation, known as Post-Activation Performance Enhancement (PAPE). This involves an acute increase in strength through dynamic exercises with a high contraction rate [22]. Acute PAPE is a frequently observed phenomenon in short-term strength-speed performance [23,24,25,26,27]. However, Blazevich and Babault [28] found that CMJ performance did not follow the typical time course of PAPE but showed improvement at all intervals from 30 s to 12 min post-load. It is known from practice that after 60–90 min of active movement, athletes can perform higher short-term speed and strength tasks [29] compared to after a short warm-up. The PAPE effect is considered a continuation of the PAP effect, resulting from improved blood perfusion to the tissue [30]. Additionally, the body may trigger other supercompensatory mechanisms, such as the general adaptation syndrome (GAS), which contribute to enhancements in both speed and strength [31]. This study evaluates the response of elite athletes and a control group to these loading conditions. Performance changes in the countermovement jump (CMJ) test, which reflects both PAPE and GAS, are analyzed in relation to the polymorphisms of selected genes, chosen based on their documented influence on tissue quality in the musculoskeletal and support systems.

The *ACE* gene, encoding angiotensin-converting enzyme, is extensively studied in sports genetics. *ACE* product converts angiotensin I to angiotensin II, also deactivates the vasodilator bradykinin, collectively resulting in increased blood pressure [32]. There are two notable *ACE* polymorphisms (rs4646994) relevant to athletic performance, the I and D alleles. The D allele is associated with hypertrophic and hyperplastic growth of cardiac and smooth muscle cells and a greater strength gain in skeletal muscles in response to physical load [33]. This allele is prevalent among power-oriented athletes [34,35,36,37] such as short-distance runners [38] and swimmers [37] and weightlifters [35] and is linked to superior performance in sprints and long jump tests [39,40]. Conversely, the I allele is more common among endurance-oriented athletes [41], who predominantly rely on aerobic metabolism. This allele is found in rowers, long-distance runners, mountaineers, and long-distance swimmers [35,42]. II homozygotes exhibit improved VO2max [43], enhanced exercise duration after training [37], and better performance in endurance tests [44]. They have a higher proportion of type I muscle fibers, greater fatigue resistance, improved peripheral oxygenation, and higher cardiac output during exercise [4,45].

The *ACTN3* gene encodes α-actinin 3, a protein expressed exclusively in type II muscle fibers. The R577X polymorphism (rs1815739) introduces a premature stop codon, reducing α-actinin-3 in homozygotes without causing pathology [46]. Research indicates that the R allele is associated with power-oriented sports, such as weightlifting [34,35,47] and sprinting [46,48,49,50]. R allele carriers typically exhibit superior performance in jump and sprint tests [40,51], higher lower extremity power [50], maximal isoinertial strength, and maximum power following resistance training [52,53,54]. The RR genotype correlated with a higher proportion of type II muscle fibers [55]. Conversely, the X allele is more frequent among endurance athletes, such as long-distance swimmers [38] and players [56]. XX genotype carriers tend to have a higher basal VO2max, lower muscle strength, and smaller muscle volume, showing a lower response to resistance training compared to RR and RX genotypes [57].

The *PPARA* gene, which codes for the peroxisome proliferator-activated receptor plays a crucial role in lipid and glucose metabolism, energy homeostasis, and body mass. The SNP polymorphism rs4253778 (7 G/C) of *PPARA* is related to athletic performance [58]. High *PPARA* expression, found in fatty acid-catabolizing tissues like the liver, skeletal muscle, and heart, is an essential factor for the adaptive response to endurance training by increasing the oxidative capacity of muscle [59,60]. The C allele may confer an advantage in power-oriented sports due to its hypertrophic effects on skeletal muscle and increased glucose utilization during anaerobic exercise [61]. Power athletes show a high frequency of the C allele, and C carriers perform better in the handgrip and the Wingate test [62,63,64]. Conversely, the GG genotype is more frequent among endurance athletes, including long-distance runners [60,63,65], rowers [66], and combat sports players [67]. The G allele correlates with higher oxygen pulse values, indicating better endurance capacity.

Adaptation to hypoxia is primarily mediated by hypoxia-inducible factor 1 (HIF-1), a transcriptional activator [68]. The *HIF1A* gene encodes the oxygen-regulated subunit HIF-1α, which is constitutively higher in glycolytic type IIX muscle fibers than oxidative type I fibers [69]. An SNP (rs11549465) in the *HIF1A* gene involves a C- to T substitution in exon 12, resulting in a proline-to-serine change at position 582. This mutation increases gene transcription activity, *HIF1A* product stability, and cell resistance to hypoxia [70]. Pro/Ser genotype carriers have a higher percentage of type IIX muscle fibers than Pro582 homozygotes [71].

Adenosine monophosphate deaminase (AMPD product) is a key regulator of muscle energy metabolism during exercise. The skeletal muscle-specific isoform of AMPD, encoded by the *AMPD1* gene, is activated after intense short-term exercise [72,73,74]. A nonsense mutation (rs17602729) in exon 2 of *AMPD1* involving a C to T transition results in a premature stop codon [75]. TT genotype carriers have reduced exercise capacity and cardiorespiratory response [76]. The CC genotype in the *AMPD1* gene benefits athletes in anaerobic performance (strength, speed, explosiveness) by enhancing regeneration, reducing muscle fatigue, and improving training response. It is ideal for short-term, high-intensity activities [72].

Volleyball players require explosive power for jumps and smashes [29,30], influenced by *ACTN3* (R) and *ACE* (D/D) [47], along with endurance, linked to *PPARA* (G) [62] and *HIF1A* [71]. Basketball players need a balance of strength, speed, and endurance, with acceleration and directional changes supported by *ACTN3* (R) [40,50,52] and *ACE* (D) [51], while endurance variants of *PPARA* [63] and *HIF1A* [71] aid performance in long matches. The *AMPD1* gene was selected for its influence on muscle regeneration after a training load.

All participants were genotyped for the selected genes. It was hypothesized that differences in absolute values before and after the training load would reveal variations in CMJ performance in elite players, associated with specific genetic polymorphisms. Based on existing knowledge of the role of individual genes and the combined effects of PAP, PAPE, and GAS, players were first tested using the countermovement jump (CMJ) before the main training load, following a brief basic warm-up during which PAP is expected to manifest. This was followed by approximately 1.5 h of high-intensity training typical of these sports. The second testing occurred 10–18 min after the completion of the main training session. A 1.5-h high-intensity load, interspersed with rest intervals, triggers immediate supercompensatory processes as part of GAS. Therefore, the post-training effect is interpreted as a combination of PAPE and GAS. By monitoring CMJ after training, we assess the impact of gene polymorphisms on performance.

## 2. Materials and Methods

### 2.1. Participants

The player group (age: 25.98 ± 5.01 years; height: 195.42 ± 7.16 cm; body mass: 91.06 ± 9.82 kg) (Table 1) consisted of 100 male athletes from the highest performance class of the Slovak Republic in the following sports disciplines: basketball (*n* = 44) and volleyball (*n* = 56). The inclusion criteria for the player group were as follows: male professional or elite athlete status (members of the wider representative selection of the Slovak Republic in basketball or volleyball), training at least four times per week, and aged 17–40. Exclusion criteria included injured or ill players and players who had not been continuously training for the last six weeks. We did not observe significant differences in age or the investigated somatic indicators between basketball and volleyball players, we combined them into a common selection of players.

The control group (age: 19.81 ± 0.75 years; height: 182.72 ± 6.53 cm; body mass: 77.89 ± 13.91 kg) (Table 1) consisted of 54 male participants (*n* = 54) from the general population with predominantly sedentary lifestyles, who engaged in physical activity only as part of compulsory physical education at the university once a week. Exclusion criteria for the control group included injury, illness, and any additional training beyond the compulsory physical education at the university. All participants were Caucasian.

Recruitment of eligible participants was conducted continuously after the approval of the ethical committee, with assistance from professional basketball and volleyball clubs and representatives or national coaches of the respective disciplines. The control group was recruited with the help of university physical education teachers. The process and purpose of the research project were explained to the participants, who subsequently agreed to participate and signed the informed consent form, which was constructed in accordance with the ethical committee’s decision.

### 2.2. Genotyping

DNA was isolated from the buccal swab using a standard protocol with a 5% Chelex solution (Bio-Rad, Hercules, CA, USA). PCR was used to detect the I and D alleles of the *ACE* gene according to the method described by Tiret et al. [77], using PCR primers as follows: forward 5′-CTGGAGACCACTCCCATCCTTTCT-3′ and reverse 5′-GATGTGGCCATCACATTCGTCAGAT-3′. This method produces a PCR fragment of 190 and 490 bp in the presence of alleles D and I, respectively. Genotypes II and DD had one band of the correspondent size, and genotype ID had two bands. Genotyping of the *ACTN3* R577X polymorphism was performed using PCR with primers designed in Primer-BLAST: forward 5′-CAGCGCACGATCAGTTCAAG-3′ corresponding to a sequence in exon 15; reverse 5′-AATCCCACGTGGAGTCTGTG-3′ corresponding to the sequence in intron 15. The amplified products of 307 bp were sequenced (Microsynth, Balgach, Switzerland). PCR was used to detect the polymorphism of the *PPARA* gene using PCR primers as follows: forward 5′-ACAATCACTCCTTAAATATGGTGG-3′; reverse 5′-AAGTAGGGACAGACAGGACCAGTA-3′, generating a fragment of 266 bp. The PCR products were sequenced (Microsynth). Genotyping of the *HIF1A* polymorphism was performed using PCR with primers designed in Primer-BLAST: forward 5′-AGGTGTGGCCATTGTAAAAACT-3′, corresponding to a sequence in intron 11; reverse 5′-AATTCATCAGTGGTGGCAGTG-3′, corresponding to a sequence in exon 12. The 255 bp PCR products were sequenced (Microsynth). PCR was used to detect the *AMPD1* gene polymorphism according to the method described by Morisaki et al. [78], using PCR primers as follows: forward 5′- GCAATCTACATGTGTCTACC-3′; reverse 5′-ATAGCCATGTTTCTGAATTA-3′, generating a fragment of 197 bp. The PCR products were sequenced (Microsynth). The volume of the PCR reaction was 20 μL, and the DNA concentration was 300–400 ng per PCR reaction. We used My Taq DNA polymerase (Bioline) and 5 × My Taq reaction buffer (Bioline, Memphis, TN, USA). The temperature and time were optimized by gradient PCR for each gene. Thermal-time PCR was as follows: initial denaturation for 8 min at 95 °C and 35 cycles with denaturation of 1 min at 95 °C, annealing for 45 s at 58 °C (*ACE*), 58.7 °C (*ACTN3* and *HIF1A*), 50 °C (*PPARA*) and 53.5 °C (*AMPD1*) and extension for 45 s at 72 °C, and final extension at 72 °C for 10 min.

### 2.3. Motor Test

The evaluation of vertical jump parameters was performed using a 10-s repeated jump test (CJ10) measured by the OptoJump Next^®^ device (Microgate, Bolzano, Italy). OptoJump Next^®^ device (Microgate, Bolzano, Italy) is an optical measurement system that determines an athlete’s performance. The OptoJump Next^®^ system (Microgate, Bolzano, Italy) demonstrates excellent reliability for estimating vertical jump height. Validity ICCs were exceptionally high (0.997–0.998), despite a consistent systematic difference from the force plate (−1.06 cm; *p* < 0.001). Test-retest reliability was also excellent, with ICCs of 0.982–0.989, a low coefficient of variation (2.7%), and minimal random error (±2.81 cm) [79]. Participants performed their jumps with their legs outstretched and their hands fixed on their hips, aiming to jump as high and take off as quickly as possible [80]. By completing jumps with outstretched legs, we aimed to prevent the effect of the stretch-shortening cycle (SSC), in which elastic abilities also play a crucial role, which we wanted to eliminate in our research due to the possibility of distorting the results [81].

The CJ10 test was performed four times: two trials before and two trials after the training unit, each with a continuous duration of 10 s. Before the first measurement, participants completed a standardized warm-up (three-minute medium-intensity run, followed by two sets of ten dynamic and jumping exercises). After warming up, they had a 5-min passive recovery period (to allow post-activation potentiation (PAP) to manifest), during which the correct jumping technique was explained to them. Following this, they underwent their first test. After training, subjects underwent a second measurement after a 15-min rest period (to allow post-activation performance enhancement (PAPE) and general adaptation syndrome (GAS) to manifest).

### 2.4. Acute Exercise

The training unit represented a 1–1.5-h specific training load, depending on the sports discipline of the player group.

The specific physical load for the volleyball players consisted of 60–150 jumps and a run of 1757 ± 462 m during the game. Basketball players, depending on their role, performed 56–69 jumps during training and ran 1.67–4.18 ± 1.95 km. Jumps were performed at submaximal to maximal intensity, and during running, anaerobic alactatic and aerobic metabolism were mostly activated. The control group had general training, which they completed as part of compulsory physical education at the university. Pre-training measurements were used to evaluate the level of basal vertical jump abilities (PAP effect). We determined the cumulative effect of PAPE and GAS after a training unit by comparing the differences between pre- and post-training measurements of the vertical jump test.

In the CJ10 test, we measured the average time of contact with the pad (tc [s]), the average height of vertical jumps (h [cm]), and the average power in the active phase of the rebound (P [W/kg]). We derived differences from the parameters of pre- and post-training measurements: ∆tc, ∆h, and ∆P.

### 2.5. Statistical Analysis

We used Chi-square analysis to compare genotype distribution between the player and control groups and Cramér’s V to measure effect size. V values of 0.10, 0.30, and 0.50 were considered small, medium, and large effects, respectively [82]. Individual participants with different genotypes were compared based on the results of the jump tests. Intragroup comparisons monitored differences based on individual genotype groups regarding tc, P, h, ∆tc, ∆P, and ∆h. To determine the normality of the data distribution, we used the Shapiro-Wilk test if *n* ≤ 50 in the subsets created based on individual genotypes, and the Kolmogorov-Smirnov test if *n* > 50 [83].

The results from comparing genotypes with the jump test parameters were statistically evaluated using the parametric statistical method one-way ANOVA, considering effect size by partial eta squared (*η*^2^). *η*^2^ values of 0.02–0.12, 0.13–0.25, and > 0.26 were considered weak, moderate, and strong associations, respectively [84]. Before ANOVA, we implemented Levene’s test for homogeneity of variances. Pearson’s correlation coefficient was used to determine the correlation between the TGS and CJ10 test values. Regarding substantive significance, we evaluated the dependence according to the interpretation of Hopkins: 0.0–0.1 (trivial), 0.1–0.3 (small), 0.3–0.5 (moderate), 0.5–0.7 (large), 0.7–0.9 (very large), and 0.9–1 (nearly perfect) [85].

Statistical processing of empirical data was carried out using IBM SPSS v23. Statistical significance was accepted at *p* ≤ 0.05 for all analyses.

## 3. Results

In our research, we tested 100 male players. We analyzed the frequency of 5 genes: *ACTN3*, *ACE*, *PPARA*, *HIF1A*, and *AMPD1* in the groups of individual players and evaluated the frequency of different combinations of genes selected alleles compared to the control population. Furthermore, in all groups tested, we measured the values of the indicators of speed-strength abilities in a 10-s continuous vertical jump test with OptoJump Next^®^ device (Microgate, Bolzano, Italy). We determined the PAP effect by comparing vertical jumps before and the PAPE and GAS effects after the specific training load. The differences in measured values were the differences (Δh, ΔP, Δtc) between the pre- and post-training measurements.

### 3.1. Frequency of Alleles and Genotypes

We compared a total of twenty-two possible genotypes. Thirteen genotypes were individually compared in 5 genes (II, ID, DD for *ACE*; RR, RX, XX for *ACTN3*; GG, GC, CC for *PARA*; Pro/Pro, Pro/Ser for *HIF1A*; CC, CT for *AMPD1*). We determined the percentage of individual genotypes and alleles among the participants in both tested groups. The distribution of genotypes was in Hardy-Weinberg equilibrium in both groups.

The genotype frequencies differed significantly between athletes and the control group only in the *ACE* and *PPARA* genes. No significant differences were observed in the frequencies of *ACTN3*, *HIF1A*, and *AMPD1* genotypes between the two groups (Table 2).

### 3.2. Indicators of Speed-Strength Abilities According to the Genotype of Selected Genes

#### 3.2.1. *ACTN3*

In terms of vertical jump height and power in the active phase of rebound, both groups had lower values in the case of genotype XX (Table 3). The participants in both groups, where even one R allele was present, showed better speed-strength abilities than the XX genotype (Table 3). In the case of ΔP, Δh differences, which were calculated from the pre-workout and post-workout values, there was an improvement in the case of players, especially in the participants with the R allele, compared to the XX genotype (Table 3). There were no significant differences between the RR and RX genotypes. In the control group, participants with the RR genotype showed higher values of ΔP and Δh differences, as participants with the RX genotype had approximately the same values of differences as participants with the XX genotype (Table 3).

#### 3.2.2. *ACE*

In the group of players, we did not find differences in the values of pre-training measurements between individual genotypes (DD, ID, II) (Table 4). In the control group, we found better jump power and height in participants with DD genotypes than ID and II genotypes, which had almost the same values (Table 4). Interesting results were observed when comparing genotypes with differences in ΔP and Δh. In the player group, participants with allele I showed improvement, with the greatest enhancement observed in those with the II genotype. In the case of the control group, the significant results of ΔP differences (1.96 W/kg) were shown by participants with homozygous genotypes (II + DD) compared to heterozygous (ID) participants, with a statistically significant result of *F* (1, 53) = 6.799, *p* = 0.012, *η*^2^ = 0.116. The genotype II groups compared to the genotype ID or DD groups, respectively, had the best values, with a significance level of *F* (2, 53) = 3.474, *p* = 0.039, *η*^2^ = 0.120 (Table 4).

#### 3.2.3. *PPARA*

In terms of the observed indicators of the vertical jump test, *PPARA* did not show a significant effect on players or controls in pre-training measurements. We found a remarkable result when comparing genotypes, as heterozygotes showed the highest positive values of ΔP (players 2.29 W/kg; control group 1.24 W/kg) and Δh differences in both players and controls (players 2.30 cm; control group 1.66 cm). We had only one participant with the CC genotype in the control group. Therefore, it is impossible to objectively assess the effect of the given genotype in terms of statistics (Table 5).

#### 3.2.4. *HIF1A*

In terms of the monitored indicators of the vertical jump test in the pre-training measurements, *HIF1A* did not show a significant effect in any group of participants. We found significant differences in the ΔP compared to *HIF1A* genotypes only in the control group, where participants with the Pro/Ser genotype had a significant improvement after training (3.08 W/kg) compared to Pro homozygotes (0.50 W/kg), *F* (1, 53) = 5.699, *p* = 0.021, *η*^2^ = 0.099, (Table 6).

#### 3.2.5. *AMPD1*

In the case of the *AMPD1* gene in players, we found a statistically significant result, where participants with the CT genotype had worse results in pre-training jumps than the CC genotype *F* (1, 99) = 4.331, *p* = 0.040, *η*^2^ = 0.042. In the control group, we found significant differences in ΔP, F (1, 53) = 14.918, *p* < 0.001, *η*^2^ = 0.223, and in Δh, F (1, 53) = 12.188, *p* = 0.001, *η*^2^ = 0.190, both indicating a moderate effect. Participants with the CT genotype even performed worse than in the pre-training measurements (−1.74 W/kg: −1.89 cm). The TT genotype did not occur in our sample. In the player group, no significant differences were found in jump parameters before and after training across individual *AMPD1* genotypes (Table 7).

## 4. Discussion

The aim of our study was to evaluate the effect of *ACE*, *ACTN3*, *PPARA*, *HIF1A*, and *AMPD1* gene polymorphisms on selected vertical jump parameters detected by the optical device OptoJump Next^®^ device (Microgate, Bolzano, Italy) at the beginning of the training unit (PAP effect) and their changes after completing the training (cumulative effect of PAPE and GAS) in a set of players (100 male players from the highest performance class in the Slovak Republic in basketball and volleyball) and a control set (54 male participants from the general population with predominantly sedentary lifestyles).

In the player group, the CT genotype of the *AMPD1* gene was associated with significantly lower vertical jump height compared to the CC genotype (*p* < 0.05), consistent with previous studies linking the T allele to reduced exercise capacity, muscle fatigue, and myopathy [42,76]. In the control group, the CT genotype was linked to significantly lower cumulative PAPE and GAS responses (ΔP, Δh) compared to CC genotype carriers, who exhibited positive adaptive responses (*p* < 0.01). The C allele, more frequent in elite athletes, is associated with enhanced speed-strength abilities [8,73]. Moreover, carriers of the TT genotype in the *AMPD1* gene demonstrate reduced exercise capacity and cardiorespiratory responses [76], with the C allele more prevalent among elite Caucasian athletes [8,73].

A significant positive effect on the power of the active phase of the vertical jump (ΔP) was observed for the II genotype of the *ACE* gene (*p* < 0.05) and the Pro/Ser genotype of the *HIF1A* gene (*p* < 0.05), both only in the control group. The Ser allele of *HIF1A* was associated with a significantly higher percentage of type IIX muscle fibers and had a higher representation in speed-strength-oriented athletes [71]. The D allele and, preferably, the DD genotype of the *ACE* gene were most often associated in previous research with better strength and speed strength indicators than carriers of the I allele [40,44,62]. They were also more represented among speed-strength-oriented athletes [35,36,42]. The C allele of the *PPARA* gene has been associated with better speed-strength abilities in several studies and is highly prevalent among speed-strength athletes [45,62,64].

Because good speed-strength abilities are essential in volleyball and basketball, we expected our players to have a higher frequency of the R allele of the *ACTN3* gene, the D allele of the *ACE* gene, the C allele of the *PPARA* gene, the Ser allele of the *HIF1A* gene, and the C allele (Gln12) of the *AMPD1* gene. The high proportion of R alleles of the *ACTN3* gene was confirmed in players, as genotypes with the R allele content (RR + RX) represented up to 86.0% (Table 2). Also, in the case of the *ACE* gene, a higher proportion of genotypes containing the D allele was confirmed since up to 76.0% of participants had the ID + DD genotype. The *PPARA* gene exhibited contrasting results, with genotypes containing the C allele accounting for only 39.0%. The GG genotype was predominant among players (61.0%), while the CC genotype was rare (7.0%). As a regulator of lipid and glucose energy metabolism during endurance exercise [60], the high prevalence of the G allele and heterozygosity in players suggests its importance in endurance-related energy pathways [62,86,87]. Despite emphasizing speed-power abilities in basketball and volleyball, our findings (Table 5) indicate the G allele’s essential role in strength endurance based on differences in P and h. In contrast, a high frequency of the CC genotype has been reported primarily in football [62,88].

Similarly, we expected better performance for carriers of the R allele of the *ACTN3* gene, the D allele of the *ACE* gene, the C allele of the *PPARA* gene, the Ser allele of the *HIF1A* gene, and the C allele (Gln12) of the *AMPD1* gene in the dynamic indicators of speed and force determined by OptoJump Next^®^ device (Microgate, Bolzano, Italy). We evaluated the effect of genotype on basal jump ability (measured before the training unit) and immediate cumulative PAPE and GAS effect (based on differences between post-training and pre-training measurements). The PAPE and GAS effect causes a load, which in our case represents one training unit.

It seemed that the R allele of the *ACTN3* gene has an advantage in basal vertical jump abilities in both players and controls compared to participants with the XX genotype. However, the results were not statistically significant. Other studies confirmed our findings, where the R allele moderately affected speed-power abilities [12,50,55]. It also seems that the R allele has a positive effect on the immediate cumulative PAPE and GAS effect, as we found better results in the post-training test compared to the pre-training test in both RR and RX genotypes compared to XX. However, even these results were not statistically significant.

The *ACE* gene had a statistically significant positive effect on the immediate cumulative PAPE and GAS effect based on the power in the active phase of the take-off. The most significant improvements occurred in the participants with genotype II in the control group. In the control group, it proves to be more advantageous to be homozygous (II, DD) than heterozygous (ID). As genotype II has been associated with better endurance, it represents an advantage in long-term physical performance, theoretically explaining our results. The DD genotype has been associated with speed-strength abilities. The main effect is through the RAAS system [33,89,90], through which blood pressure increases, improving blood flow to the working tissue. Angiotensin II, part of the RAAS system, also has the function of a growth factor that increases muscle volume and strength and increases the percentage of type II fast glycolytic muscle fibers in muscles [33,89]. This is likely to be an advantage for untrained individuals. In players with a higher level of training, the advantage of the D allele likely diminishes, while the I allele appears to confer a benefit for endurance abilities. Another possible explanation may be that the ID genotype does not represent a dominant genotype for either phenotype—a non-dominant, mixed genotype. In this case, other genes with a more significant effect on speed strength abilities may manifest.

In the case of the *PPARA* gene, we did not find statistically significant results. A statistically significant effect of the *HIF1A* gene was captured in the control group on the cumulation effect of PAPE and GAS based on ΔP values. The Pro/Ser genotype positively affected the immediate cumulation effect of PAPE and GAS. However, this effect was not found in the player group. The Pro/Pro genotype seemed to have a positive effect, even though the result was not statistically significant in the player group. According to other publications, individuals with the Pro/Ser genotype had an advantage for anaerobic and lactate metabolism compared to the Pro/Pro genotype [71]. We observed a statistically significant difference associated with the basal jump abilities in the player group in the case of the *AMPD1* gene, where the CT genotype represented a disadvantage in vertical jump height compared to the CC genotype. Different genotypes of the *AMPD1* gene had the same cumulation effect on PAPE and GAS in players. However, in the control group, the CT genotype had a statistically significant disadvantage compared to the CC genotype, as participants with the CT genotype had negative ΔP and Δh compared to positive values of differences in both indicators in carriers of the CC genotype. This disadvantage is consistent with the results of [45], in which the T allele is associated with the most frequent myopathy in the population. This means that players with the T allele cannot achieve results similar to those without the T allele. Our results indicate that the influence of genetic polymorphisms of the *ACE*, *HIF1A*, and *AMPD1* genes on basal jump ability and cumulative PAPE and GAS effect depends on the participants’ training adaptation level. Research has direct implications for both coaches and the scientific community. For coaches, it highlights the practical application of gene polymorphisms in optimizing training stimuli based on individual genotypes. This approach enables the strategic utilization of players according to their suitability for strength-speed demands and their effectiveness over partial or full match durations. For the scientific community, the study provides insights into the distribution of relevant genotypes among elite athletes in the Central European region, offering a foundation for further research in this field.

## 5. Conclusions

The II genotype of the *ACE* gene and the Pro/Ser genotype of the *HIF1A* gene positively affect the immediate cumulation of PAPE and GAS in individuals who are not accustomed to training. Conversely, the CT genotype of the *AMPD1* gene negatively impacts the accumulation of PAPE and GAS in individuals who are not adapted to training. Additionally, the CT genotype adversely affects basal vertical jump performance in training-adapted players. The polymorphisms of the *ACTN3* and *PPARA* genes have no statistically significant effect on jump abilities or cumulation PAPE and GAS. In conclusion, in a trained individual, the influence of genotype is suppressed by the cumulation PAPE and GAS effect, by which the organism adapts to the stress caused by training and competition loads. Adaptation increases the conditioning abilities required for elite performance. Our findings show that different gene polymorphisms have a variable effect on the combined effect of PAPE and GAS, which is reflected in vertical jump parameters, depending on the participants’ training adaptation level.

Limitations: The primary limitations of our study involve working with groups of elite athletes who were selected for teams based on their game performance, with physical performance being a secondary monitored parameter. We could not ensure an identical movement load during the training sessions for all participants. Furthermore, because the participants were male professional or elite athletes, we were unable to make substantial modifications to their training regimens. The sample size and the inclusion of only one gender were determined by the availability of elite athletes, all of whom were either national team members or played for top three teams in the national league. We acknowledge the study’s limitation in examining a restricted set of genes and the potential influence of gene interactions on phenotypic traits. Future research should therefore expand the scope to include additional gene polymorphisms known to influence motor skill development in sports.

Future lines: In subsequent research, a greater number of genes influencing the development of locomotor abilities should be monitored, considering the potential complexity of gene interactions related to the locomotor apparatus. In the second phase, the potential impact on common injuries among elite athletes will be examined. This phase will involve a broader age range of subjects and will incorporate orthopedic and traumatological follow-ups.

Strength of our data: All measurements were conducted by the same group of examiners under consistent and identical conditions. For instance, the measuring device was always placed at the strongest possible point, no more than 120 cm from the hall wall, to minimize the influence of floor flexibility. All participants received thorough instructions and were required to practice the measurement protocol several times. Elite athletes had already routinely used this measurement in their training activities for an extended period.

## Figures and Tables

**Table 1:** Descriptive statistics of participant characteristics.

	Player Group (*N* = 100)	Control Group (*N* = 54)	*p*-Values
	Mean ± *SD* (95% CI)	Mean ± *SD* (95% CI)	
Age (years)	25.98 ± 5.01 (16.16; 35.80)	19.81 ± 0.75 (18.34; 21.29)	<0.001
Body height (cm)	195.42 ± 7.16 (181.40; 209.44)	182.72 ± 6.53 (169.92; 195.53)	<0.001
Body mass (kg)	91.06 ± 9.82 (71.82; 110.30)	77.89 ± 13.91 (50.63; 105.15)	<0.001

Notes. *N*—quantity, *SD*—standard deviation.

**Table 2:** Frequency of genotypes in the player and control groups.

		Player Group (*n* = 100)	Control Group (*n* = 54)		Player Group (*n* = 100)		Control Group (*n* = 54)		*p*-Values
Gene	Allele			Genotype	*N*	%	*N*	%	
*ACTN3*				RR	45	45.0	20	37.0	
	R	65.5	59.3	RX	41	41.0	24	44.5	0.212
	X	34.5	40.7	XX	14	14.0	10	18.5	
*ACE*				DD	35	35.0	13	24.1	
	D	55.5	53.7	ID	41	41.0	32	59.2	**0.001**
	I	44.5	46.3	II	24	24.0	9	16.7	
*PPARA*				CC	7	7.0	1	1.9	
	G	77	85.2	GC	32	32.0	14	25.9	**<0.001**
	C	23	14.8	GG	61	61.0	39	72.2	
*HIF1A*	Pro	93.5	93.5	Pro/Pro	87	87.0	47	87.0	1
	Ser	6.5	6.5	Pro/Ser	13	13.0	7	13.0	
*AMPD1*	C	89	89.8	CC	78	78.0	43	79.6	0.691
	T	11	10.2	CT	22	22.0	11	20.4	

Note. *n*—quantity.

**Table 3:** Descriptive statistics of vertical jump parameters by *ACTN3* genotypes in player and control groups.

	Player Group			Control Group		
	RR (*n* = 45)	RX (*n* = 41)	XX (*n* = 14)	RR (*n* = 20)	RX (*n* = 24)	XX (*n* = 10)
tc (s)	0.190 (±0.020)	0.187 (±0.017)	0.199 (±0.032)	0.216 (±0.039)	0.216 (±0.040)	0.236 (±0.030)
P (W/kg)	50.16 (±10.23)	49.84 (±8.30)	46.16 (±10.70)	36.38 (±7.15)	38.80 (±8.07)	32.89 (±7.23)
h (cm)	37.01 (±6.84)	36.94 (±5.47)	34.41 (±5.66)	27.68 (±4.46)	29.59 (±5.14)	26.03 (±4.96)
∆tc (s)	0.002 (±0.011)	0.003 (±0.012)	0.000 (±0.015)	0.004 (±0.015)	0.000 (±0.015)	0.001 (±0.018)
∆P (W/kg)	1.95 (±2.69)	1.86 (±2.77)	1.54 (±3.99)	0.98 (±2.87)	0.76 (±2.63)	0.72 (±3.23)
∆h (cm)	1.82 (±1.77)	2.29 (±1.93)	1.79 (±2.33)	1.30 (±3.54)	0.60 (±2.91)	1.00 (±3.97)

Notes. *n*—quantity, tc—contact time, P—power in the active phase of the take-off, h—jump height, ∆tc—the difference between the post-training and pre-training contact times, ∆P—the difference between the post-training and pre-training power in the active phase of the take-off, ∆h—the difference between the post-training and pre-training jump heights, values are means, values in parentheses represent standard deviation (±SD).

**Table 4:** Descriptive statistics of vertical jump parameters by *ACE* genotypes in player and control groups.

	Player Group			Control Group		
	DD (*n* = 35)	ID (*n* = 41)	II (*n* = 24)	DD (*n* = 13)	ID (*n* = 32)	II (*n* = 9)
tc (s)	0.188 (±0.024)	0.190 (±0.021)	0.193 (±0.015)	0.224 (±0.046)	0.216 (±0.034)	0.229 (±0.042)
P (W/kg)	49.77 (±9.82)	49.18 (±10.08)	49.54 (±8.56)	38.33 (±9.07)	36.52 (±7.45)	35.62 (±7.39)
h (cm)	36.68 (±6.27)	36.26 (±6.17)	37.14 (±6.19)	29.44 (±5.64)	27.91 (±5.04)	27.60 (±3.57)
∆tc (s)	0.004 (±0.012)	0.001 (±0.012)	0.001 (±0.013)	0.001 (±0.016)	0.003 (±0.015)	0.003 (±0.016)
∆P (W/kg)	1.41 (±3.45)	2.00 (±2.43)	2.26 (±2.80)	1.73 (±2.62)	0.06 (±2.77)	**2.30 a** (±2.25)
∆h (cm)	2.01 (±2.10)	1.92 (±1.99)	2.16 (±1.53)	1.72 (±3.11)	0.23 (±3.58)	2.32 (±1.80)

Notes. *n*—quantity, tc—contact time, P—power in the active phase of the take-off, h—jump height, ∆tc—the difference between the post-training and pre-training contact times, ∆P—the difference between the post-training and pre-training power in the active phase of the take-off, ∆h—the difference between the post-training and pre-training jump heights, values are means, values in parentheses represent standard deviation (±SD), **a**—*p* = 0.039 significant differences between participants from the control group with different *ACE* genotypes (DD vs. II vs. ID).

**Table 5:** Descriptive statistics of vertical jump parameters by *PPARA* genotypes in player and control groups.

	Player Group			Control Group		
	CC (*n* = 7)	GC (*n* = 32)	GG (*n* = 61)	CC (*n* = 1)	GC (*n* = 14)	GG (*n* = 39)
tc (s)	0.188 (±0.021)	0.194 (±0.023)	0.189 (±0.020)	0.205	0.223 (±0.046)	0.219 (±0.036)
P (W/kg)	48.09 (±10.07)	49.70 (±9.60)	49.51 (±9.62)	52.54	35.91 (±6.49)	36.73 (±7.92)
h (cm)	35.44 (±5.26)	36.90 (±6.40)	36.60 (±6.19)	40.38	27.17 (±3.17)	28.29 (±5.16)
∆tc (s)	0.004 (±0.016)	0.000 (±0.011)	0.003 (±0.012)	0.017	0.004 (±0.011)	0.001 (±0.017)
∆P (W/kg)	1.30 (±3.13)	2.29 (±3.04)	1.70 (±2.82)	−3.34	1.24 (±2.44)	0.79 (±2.87)
∆h (cm)	1.67 (±2.20)	2.30 (±2.09)	1.90 (±1.81)	−2.41	1.66 (±2.34)	0.76 (±3.59)

Notes. *n*—quantity, tc—contact time, P—power in the active phase of the take-off, h—jump height, ∆tc—the difference between the post-training and pre-training contact times, ∆P—the difference between the post-training and pre-training power in the active phase of the take-off, ∆h—the difference between the post-training and pre-training jump heights, values are means, values in parentheses represent standard deviation (±SD).

**Table 6:** Descriptive statistics of vertical jump parameters by *HIF1A* genotypes in player and control groups.

	Player Group		Control Group	
	Pro/Pro (*n* = 87)	Pro/Ser (*n* = 13)	Pro/Pro (*n* = 47)	Pro/Ser (*n* = 7)
tc (s)	0.191 (±0.022)	0.188 (±0.012)	0.220 (±0.033)	0.222 (0.068)
P (W/kg)	49.30 (±9.47)	50.64 (±10.40)	36.80 (±7.55)	36.84 (9.72)
h (cm)	36.41 (±5.78)	38.02 (±8.40)	28.26 (±5.12)	27.96 (4.12)
∆tc (s)	0.002 (±0.012)	0.002 (±0.012)	0.001 (±0.015)	0.007 (±0.017)
∆P (W/kg)	1.93 (±2.87)	1.35 (±3.15)	0.50 (±2.58)	**3.08 a** (±3.29)
∆h (cm)	2.00 (±1.93)	2.10 (±1.88)	0.69 (±3.20)	2.59 (±3.83)

Notes. *n*—quantity, tc—contact time, P—power in the active phase of the take-off, h—jump height, ∆tc—the difference between the post-training and pre-training contact times, ∆P—the difference between the post-training and pre-training power in the active phase of the take-off, ∆h—the difference between the post-training and pre-training jump heights, values are means, values in parentheses represent standard deviation (±SD), **a**—*p* = 0.021 significant differences between participants from the control group with different *HIF1A* genotypes (Pro/Pro vs. Pro/Ser).

**Table 7:** Descriptive statistics of vertical jump parameters by *AMPD1* genotypes in player and control groups.

	Player Group		Control Group	
	CC (*n* = 78)	CT (*n* = 22)	CC (*n* = 43)	CT(*n* = 11)
tc (s)	0.190 (±0.022)	0.192 (±0.017)	0.218 (±0.041)	0.228(±0.026)
P (W/kg)	50.36 (±9.45)	46.32 (±9.44)	37.14 (±7.15)	35.53(±10.09)
h (cm)	37.29 (±6.08)	**34.25 a** (±5.95)	28.28 (±4.45)	27.99(±6.90)
∆tc (s)	0.003 (±0.012)	0.000 (±0.012)	0.003 (±0.016)	−0.002(±0.012)
∆P (W/kg)	1.77 (±3.06)	2.16 (±2.31)	1.49 (±2.52)	**−1.74 b**(±2.27)
∆h (cm)	1.98 (±2.00)	2.12 (±1.65)	1.66 (±3.12)	**−1.89 c**(±2.50)

Notes. *n*—quantity, tc—contact time, P—power in the active phase of the take-off, h—jump height, ∆tc—the difference between the post-training and pre-training contact times, ∆P—the difference between the post-training and pre-training power in the active phase of the take-off, ∆h—the difference between the post-training and pre-training jump heights, values are means, values in parentheses represent standard deviation (±SD), **a**—*p* = 0.040 significant differences between participants from the player group with different *AMPD1* genotypes (CC vs. CT), **b**—*p* < 0.001 significant differences between participants from the control group with different *AMPD1* genotypes (CC vs. CT), **c**—*p* = 0.001 significant differences between participants from the control group with different *AMPD1* genotypes (CC vs. CT).

## Data Availability

The original contributions presented in this study are included in the article. Further inquiries can be directed to the corresponding authors.
